# The Role of Minocycline in the Treatment of Nosocomial Infections Caused by Multidrug, Extensively Drug and Pandrug Resistant *Acinetobacter baumannii:* A Systematic Review of Clinical Evidence

**DOI:** 10.3390/microorganisms7060159

**Published:** 2019-06-01

**Authors:** Paraskevi C. Fragkou, Garyfallia Poulakou, Andromachi Blizou, Myrto Blizou, Vasiliki Rapti, Drosos E. Karageorgopoulos, Despoina Koulenti, Antonios Papadopoulos, Dimitrios K. Matthaiou, Sotirios Tsiodras

**Affiliations:** 1 4th Department of Internal Medicine, Attikon University Hospital, National and Kapodistrian University of Athens; 12462 Athens, Greece; mahiblizou@hotmail.com (A.B.); myrto_bl@hotmail.com (M.B.); vassiarapti@gmail.com (V.R.); drkarag@gmail.com (D.E.K.); antpapa1@otenet.gr (A.P.); 2 3rd Department of Medicine, Sotiria General Hospital, National and Kapodistrian University of Athens, 11527 Athens, Greece; gpoulakou@gmail.com; 3 Adult Critical Care Unit, Attikon University Hospital, National and Kapodistrian University of Athens, 12462 Athens, Greece; d.koulenti@uq.edu.au (D.K.); d.matthaiou@gmail.com (D.K.M.); 4 BTCCRC, UQCCR, Faculty of Medicine, University of Queensland, Brisbane, QLD 4072, Australia

**Keywords:** minocycline, nosocomial infections, *Acinetobacter baumannii*, antimicrobial drug resistance, MDR, XDR, PDR

## Abstract

Treatment options for multidrug resistant *Acinetobacter baumannii* strains (MDR-AB) are limited. Minocycline has been used alone or in combination in the treatment of infections associated with AB. A systematic review of the clinical use of minocycline in nosocomial infections associated with MDR-AB was performed according to the PRISMA-P guidelines. PubMed-Medline, Scopus and Web of Science ^TM^ databases were searched from their inception until March 2019. Additional Google Scholar free searches were performed. Out of 2990 articles, 10 clinical studies (9 retrospective case series and 1 prospective single center trial) met the eligibility criteria. In total, 223 out of 268 (83.2%) evaluated patients received a minocycline-based regimen; and 200 out of 218 (91.7%) patients with available data received minocycline as part of a combination antimicrobial regimen (most frequently colistin or carbapenems). Pneumonia was the most common infection type in the 268 cases (80.6% with 50.4% ventilator-associated pneumonia). The clinical and microbiological success rates following minocycline treatment were 72.6% and 60.2%, respectively. Mortality was 20.9% among 167 patients with relevant data. In this systematic review, minocycline demonstrated promising activity against MDR-AB isolates. This review sets the ground for further studies exploring the role of minocycline in the treatment of MDR-AB associated infections.

## 1. Introduction

Minocycline, semi-synthetic derivative of tetracycline, initially introduced in the 1960s, is a broad-spectrum antibacterial agent with activity against both aerobic and anaerobic gram-positive and gram-negative microorganisms [1,2,3]. Its antimicrobial properties are a result of protein synthesis inhibition and thus it exhibits a bacteriostatic effect. When administered orally, it is readily bioavailable as it is rapidly absorbed by the gastrointestinal tract (95–100%) [2,3,4,5]. These properties make minocycline a potential candidate for switching from an intravenous to an oral formulation without compromising its effectiveness, which may reduce the length and the cost of hospitalization, although studies to support this hypothesis are needed. Minocycline’s longevity in clinical practice provides the advantage of a well-documented safety profile with an overall adverse event incidence rate of 13 per million per year between 1966 and 2003 [6]. However, prolonged use has been associated with hepatotoxicity, photosensitivity, irreversible skin discolouration and autoimmune phenomena, like drug-induced systemic lupus-erythematosus [2].

The era of injudicious use of antibiotics has led to a multidrug resistant (MDR) microbial clone selection, especially in the healthcare settings, thus leaving limited therapeutic options for nosocomial infections. The Infectious Diseases Society of America (IDSA) has voiced concerns, particularly for a group of resistant bacteria, the-so-called ESKAPE pathogens (*Enterococcus faecium, Staphylococcus aureus, Klebsiella pneumoniae, Acinetobacter baumannii, Pseudomonas aeruginosa* and *Enterobacter* species) [7,8,9]. Among them, *Acinetobacter baumannii* is a strictly aerobic gram-negative non-lactose-fermenter coccobacillus, member of the *Acinetobacter calcoaceticus-baumannii complex* (ACB). A broad variety of nosocomial infections are attributable to *A. baumannii* such as bacteremia, hospital-acquired pneumonia (HAP) and ventilator-associated pneumonia (VAP), wound and soft tissue infections (SSTIs), meningitis and urinary tract infections, occurring mainly in critically ill hosts [10,11,12,13].Although its virulence has been a matter of debate, an association with crude mortality rates as high as 68% has been reported for *Acinetobacter spp*. [14,15,16,17,18,19]. The organism’s remarkable advantage of surviving for extensive periods under different environmental conditions on hospital surfaces, may underlie its ability to cause nosocomial outbreaks [20]. *A. baumannii* has evolved to become one of the most important multidrug resistant organisms (MDROs), with recent nosocomial clones exhibiting a resistance against many classes of antimicrobials by genomic mutations in topoisomerases and the up-regulation of the efflux pumps expression, production of oxacillinases like OXA-23, -24 and -58, Verona-integron-encoded metallo-β-lactamases (VIM) and New-Delhi MBL (NDM-1), as well as outer membrane porin changes [21,22,23].

The treatment options of MDR, extensively drug resistant (XDR) and pandrug resistant (PDR) *A. baumannii* infections are considerably limited. Currently, the golden standard for the treatment of infections caused by *A. baumannii* is carbapenems due to their intrinsic activity against this pathogen [24]. Polymyxins (polymyxin E or colistin and polymyxin B) also exhibit good activity and retain low but rising resistance rates among *A. baumannii* isolates [24,25,26]. As a result of the increasing resistance and the paucity of novel antibiotics, few treatment options have been left in the clinicians’ armamentarium to confront with a continuously growing healthcare issue. As novel antimicrobial agents are yet to be developed, reinstituting the use of older antimicrobials has now become a priority. The role of an old antibiotic like minocycline in the treatment of *A. baumannii* is still being explored. The favourable safety profile and the relatively low cost, make minocycline an attractive therapeutic option. Hence, a systematic review of current data is imperative in order to codify a unifying frame of practice for the use of minocycline in the treatment of MDR *A. baumannii* infections.

## 2. Materials and Methods 

The study protocol of this systematic review was designed according to the PRISMA-P (Preferred Reporting Items for Systematic Reviews and Meta-Analyses) guidelines [27,28]. The PRISMA-P checklist is availablein Table S1.

### 2.1. Inclusion Criteria

A study of any design was considered as eligible if it fulfilled all of the following criteria: (i) it included patients aged ≥ 18 years old, (ii) with nosocomial infections, (iii) caused by MDR and/or XDR and/or PDR *A. baumannii* (or *Acinetobacter calcoaceticus*-*baumannii* complex), (iv) it reported the utilization of minocycline, either in an oral or in intravenous formulation, alone or in combination with other antimicrobial agents, and (v) it designated the clinical and/or the microbiological success of the minocycline or the minocycline combinations either as a primary outcome or as a secondary outcome. No restrictions in terms of: site of infection, sample size, minocycline doses, route of administration, use of minocycline combinations, publication status, country or language were applied.

### 2.2. Exclusion Criteria

Studies were excluded if: (i) they included non-adult patients or included both adult and non-adult participants but without separate analysis for adult patients, (ii) they included community acquired or both nosocomial and community acquired infections, but without separate analysis for nosocomial infections, (iii) the isolates were not specified as *baumannii* or *A. calcoaceticus-baumannii* complex, (iv) the strains were not reported as MDR and/or XDR and/or PDR, (v) they did not concisely report that *A. baumannii* or *A. calcoaceticus-baumannii* complex strains were the causative organisms of the nosocomial infections and not just colonization isolates, (vi) no minocycline was administered, (vii) they were literature or systematic reviews, published conference abstracts or book chapters (viii) the full text could not be retrieved and, (ix) they were published in a language other than English, and a translation was technically impossible or unsound.

### 2.3. Definitions and Assumptions

The definitions and assumptions that were utilized in this systematic review are shown in Table 1. However, the definitions similar but not identical with the aforementioned ones used in individual studies were also acceptable.

### 2.4. Search Strategy

PubMed-Medline, Scopus and Web of Science^TM^ were searched from their inception until the 20th of March 2019. The search terms that were used were: “minocycline”, “acinetobacter baumannii”, “gram-negative” and “resistant”, used in the search string as follows: (“minocycline” AND (“acinetobacter baumannii” OR “gram-negative”) AND “resistant”), applied to all search fields. For the Scopus database, the search string was: ALL (“minocycline” AND (“acinetobacter baumannii” OR “gram-negative”) AND “resistant”)). Hand search of studies included in previously published systematic reviews as well as free Google Scholar searches were performed in order to identify possibly includable trials that were not identified in the initial database search.

### 2.5. Outcomes & Prioritization

The designated outcomes of the included studies regarding nosocomial infections caused by MDR, XDR or PDR *A. baumannii* strains treated with minocycline were primarily the clinical success and microbiological cure as they were defined in the “Definitions” section. Secondary outcomes that were collected in patients who received minocycline, and when available in the group of the other administered antimicrobials, were: route, duration and doses of antibiotics, duration of hospitalization (in days), mortality rate, requirement for readmission to hospital (where an adequate follow-up was obtained), reported adverse events related to minocycline or minocycline combinations, and reported adverse events to other antibiotics.

### 2.6. Risk of Bias in Individual Studies &Confidence in Cumulative Evidence

Eligible clinical studies were assessed for their quality and possible bias against the STROBE (Strengthening the Reporting of Observational Studies in Epidemiology) guidelines [31].

### 2.7. Data Synthesis &Statistical Analysis

The data collected from the retrieved studies were tabulated, grouped and consequently analyzed through an integrative synthesis method. The grouping of patients was based on the administered treatment.

## 3. Results

### 3.1. Search Results

**Figure 1:**
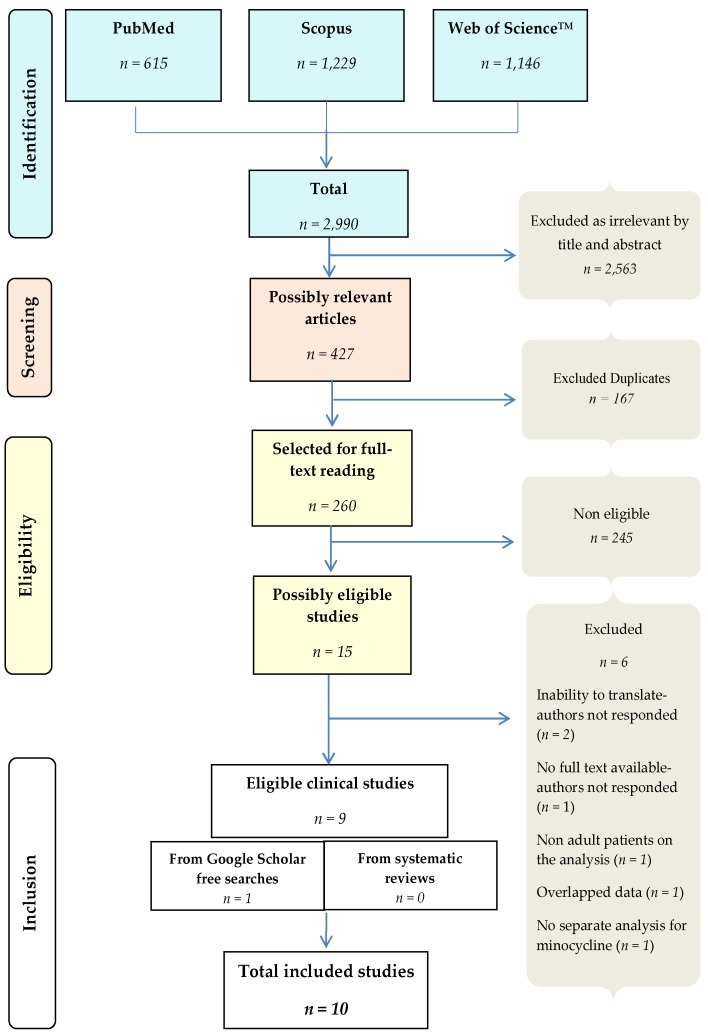
Systematic review flow chart.

The systematic search revealed 10 clinical studies eligible for inclusion: 9 studies from databases’ systematic search and 1 study from Google Scholar free search. No additional eligible studies for inclusion were identified by the manual search of previously published systematic reviews. Figure 1 shows the selection process of the included studies.

### 3.2. Study Range and Characteristics

The 10 clinical studies satisfying all the inclusion and none of the exclusion criteria are shown in Table 2. STROBE criteria for each study are demonstrated in Table A1. For the sake of brevity, each study was assigned an “Abbreviation ID” title from *A* to *J* which will henceforth be utilized instead of the study’s first author’s name and title. In general, all but two studies were published in English. Studies *G* and *H* were written in Chinese, so a manual translation was carried out. Six studies were undertaken in different regions of United States, one study in Argentina, while the rest of them were performed in China (studies *E, G* and *H*). All studies are dated from 2005 onwards, except study *I* which was done in 1998. No randomized controlled trials were identified. Four out of 10 studies evaluated the outcomes of patients with nosocomial infections caused by resistant *A. baumannii* strains treated with minocycline or minocycline combinations (studies *C, D, E* and *G*), studies *A* and *F* reported outcomes from patients that received minocycline for different pathogens, including *A. baumannii* strains, whereas the remaining studies examined the clinical effects of different antimicrobial agents in *A. baumannii* infections. Four studies were performed in Intensive Care Unit (ICU) patients, one study included patients from both the general wards and ICU, one from a burn department, whilst the rest of them did not define the patients’ origins.

In total, 286 patients were included in these studies. Among these patients, 268 (93.7%) were diagnosed with *A. baumannii* nosocomial infections, thus representing the sample size of interest for this systematic review. These patients were treated with minocycline monotherapy, minocycline combinations or other antimicrobial agents. The remaining 18 patients were reported as primarily diagnosed with MRSA or Enterobacteriaceae infections. The age range of the patients with *A. baumannii* infections was 18–106 years old. 152 out of the221 patients with available demographic data (68.8%) were men. 

The isolated pathogens represented a variety of resistant *A. baumannii* strains, with diversity in the definitions used by the authors (Table 3). Although the majority of the definitions are not entirely in concordance with the definitions proposed by Magiorakos et al., all but 2 studies have included strains with “at least” an MDR profile. In total, carbapenem-resistant (CR), MDR, XDR and PDR *A. baumannii* strains were isolated from 62 (23.1%), 78 (29.1%), 77 (28.7%) and 51 (19%) patients respectively, according to the resistance profile given by the authors.

Documented *A. baumannii* infections treated either with a minocycline-based regimen or with other antimicrobial agents are shown in Table 3. The vast majority of these infections were pneumonias (n = 216, 80.6%), 5 with concomitant bacteremia and 4 with concomitant skin structure infections. 109 (50.4%) patients were mechanically ventilated and treated for VAP. In addition, 19 (7.1%) patients were treated for bloodstream infections, osteomyelitis and prosthetic joint infection were documented in 13 (4.9%) patients, 6 (2.2%) patients had wound/surgical site/SSTIs and another 6 (2.2%) patients were diagnosed with complicated intra-abdominal infections (cIAIs). Multiple sites (≥3) were documented in 10 (3.7%) patients, 4 of whom had pneumonia, bacteremia and a wound infection simultaneously. Finally, one study reported two episodes of complicated urinary tract infection (study *C*).

In total, 223 (83.2%) patients received minocycline as a monotherapy or in combination (Table 3), whereas the remaining 45 (16.8%) were treated with other agents. In the minocycline group, monotherapy and combination was administered in 18 (8.3%) and 200 (91.7%) out of 218 patients with the available data, respectively. The dose of minocycline in most studies was 100 mg twice daily (either in oral or in intravenous formulation) with or without a loading dose of 200 mg, with the exception of studies *E* and *F* where the doses were higher (200 mg four times and two times per day, respectively). The reason of the high dose administration was not sufficiently justified by the authors. The most frequent antimicrobials used in combination with minocycline were colistin (intravenous or inhaled), polymyxin B, cefoperazone/sulbactam and carbapenems (meropenem, doripenem and imipenem +/− cilastatin). From the 200 patients on a minocycline - based combination regimen, 26 (13%) received also colistin or polymyxin B; 14 (7%) were on a colistin plus a carbapenem combination; cefoperazone/sulbactam was combined with minocycline in 90 patients (45%) whereas in 7 (3.5%) patients minocycline was combined with a carbapenem. Finally, 16 (8%) were treated with a carbapenem plus cefoperazone/sulbactam plus minocycline. The remaining 47 (23.5%) patients received a minocycline combination with other antimicrobials. The majority of the subjects were on a sulbactam-based combination (*n* = 124, 62%). From the provided data, the duration of the minocycline administration varied significantly between the studies, with a range of 2 days to 3.3 months depending on the site of infection. 

Non-minocycline based treatments were administered in 45 subjects. All patients were treated for *A. baumannii* associated VAP, except one patient who suffered from concurrent *A. baumannii* VAP and wound infection and one patient with a prosthetic joint infection. The most frequently administered antibiotics were aminoglycocides, either alone or in combination (13 out of 23 patients with the available data, 56.5%).

### 3.3. Effect of Intervention on Outcomes

Although the results in terms of outcomes were documented in all studies, the definitions of clinical and microbiological success varied across the studies (Table 3).

The outcomes were divided into two major groups: the group of patients who received a minocycline-based regimen either as monotherapy or in combination with other antimicrobials (“minocycline group”) and the group where no minocycline was administered (“other antimicrobials group”) and are summarized in Table 4.

In the minocycline group (*n* = 223), the overall reported clinical success rate was 72.6% (*n* = 162) for all documented infections. The clinical success rates ranged between 62.3% and 100% among the individual studies; the highest rates were reported in three small USA studies and one study from Argentina, whereas the lower ones were documented in two Chinese studies. A clinical cure was documented in 16 out of 18 (88.9%) and in 141 out of 200 (70.5%) patients (with available data) who received minocycline monotherapy and combination therapy respectively. Microbiological data were available for 171 (76.7%) patients in the minocycline group (*n* = 223); among them, 103 (60.2%) had a documented or presumed microbiological success.

Lower respiratory tract infection was the most prevalent type of infection. In total, 172 out of 216 (79.6%) pneumonias (with or without another concomitant *A. baumannii* infection) were treated with a minocycline-based regimen. Clinical success was reported in 121 (70.3%) patients. For the 65 minocycline- treated VAPs in particular, the success rate was 78.5% (*n* = 51). Additionally, from 28 documented bloodstream infections (alone or combined with other sites of infection) in the minocycline group, a clinical cure was obtained in 22 patients (78.6%). Clinical success was reported in 22 out of 25patients (88%) with osteomyelitis, prosthetic joint infections and/or SSTIs (alone or combined with other sites of infection) who received minocycline either as a monotherapy or in combination with other antimicrobials. Mortality rates were available in 8 studies. From 167 patients with relevant data, 35 patients died resulting in a mortality rate of 20.9%. All deaths were clinically judged as being associated with the primary infection and, therefore, they were considered as clinical failures of the minocycline. Finally, the data for hospital readmissions were not available in any of the evaluated studies.

In the non-minocycline group, a clinical cure was obtained in 21 (46.7%) patients. All infections in this group were VAP, except in study *J* that included one prosthetic joint infection. A microbiological cure was reported in 5 out of 26 (19.2%) with available data.

Adverse events in the minocycline group were reported in 9 patients (4%). Namely, 2 patients experienced an acute kidney injury likely attributable to the co-administered colistin. One patient developed neutropenia and eosinophilia whilst on the minocycline treatment. Importantly, an increase of liver function tests (LFTs) and abnormally high coagulation times was observed in 6 subjects from study *G* that included patients with cIAIs.

## 4. Discussion

This review aimed to systematically evaluate the existing evidence on minocycline effectiveness in the treatment of nosocomial infections caused by MDR, XDR and PDR *A. baumannii* strains. In brief, among 268 adult patients with a variety of nosocomial infections caused by MDR, XDR or PDR *A. baumannii* strains, 223 patients received a minocycline-based regimen with an overall documented clinical success rate of 72.6%. The majority of the patients received a minocycline combination, with a clinical success rate of 70.5%, compared to 88.9% in the monotherapy counterpart. The most prevalent infection in this study population was lower respiratory tract infection, including VAP (80.6%). The observed clinical cure rate among pneumonias was 70.3%, whereas for VAP in particular, the reported success rate was 78.5%. The microbiological success rate was 60.2%, and the crude mortality rate in the minocycline group was 20.9%. The results of the current systematic review suggest that minocycline may be an effective antimicrobial agent in the treatment of resistant *A. baumannii* nosocomial infections. However, it would be risky to draw robust conclusions through direct comparisons as no relevant adjustments have been made for the two evaluated groups of treatment. In this review, 91.7% of the participants received a minocycline combination with one or more classes of other antimicrobial agents. It is quite interesting that minocycline monotherapy demonstrated higher percentages of clinical success compared to combination regimens. Whether the administration of combinations occurred in more severe underlying infections or sicker patients and therefore resulted in lower success rates, or indeed, whether minocycline is more effective as a monotherapy, cannot be answered from this data. Many microbiological studies have reported the synergistic effects of minocycline when combined with other agents like rifampicin, imipenem, cefoperazone/sulbactam or colistin in in vitro experiments [42,43,44,45]. Similarly, in animal studies, combination regimens with rifampicin, amikacin or polymyxin B have also demonstrated promising results against experimental *A. baumannii* infections [46,47].

As opposed to the in vitro and animal data, the evidence for the advantages of combination therapies over monotherapies in the treatment of MDROs in clinical practice is not vigorous and is still being explored [48]. A meta-analysis of colistin monotherapy versus colistin combinations in the treatment of carbapenem resistant gram-negative bacteria reported that the subgroups of colistin/carbapenem, colistin/rifampicin and colistin/tigecycline did not demonstrate any benefits in mortality over monotherapy [49]. Correspondingly, a systematic review of combination therapies versus monotherapies for MDR, XDR and PDR *A. baumannii* infections was rather inconclusive in terms of superiority between the two therapeutic approaches [50]. However, the use of combinations does not come without consequences. The utilization of possibly non-effective antimicrobials in therapeutic schemes, based on in vitro synergistic effects, may induce or maintain colonization with more resistant bacteria, contributes to *Clostridium difficile* and fungal infections and increases the antibiotic-related adverse events [49,51]. 

On examining the clinical outcomes of this review, a diversity of clinical success rates is observed between two Chinese and the remaining studies, with the former ones reporting the lowest success rates. Whether this could be attributable to the advanced resistance profile of their isolates or just a confounder of the patient populations’ baseline characteristics, is not clear. Nonetheless, the regional use of minocycline in each country and its consequential impact on the susceptibility rates should be taken into account. The global data suggest that resistance has been continuously rising, even for last-resort antibiotics like polymyxins; thus, the total stock of antibiotic effectiveness steadily declines [52]. Sensibly, the pattern of microbial resistance between and within the countries is reflecting the patterns of the antibiotic use and the regional infectious diseases [52]. Therefore, it may be reasonable to say that minocycline susceptibility patterns would follow the same norm, whereby countries with a higher consumption would exhibit higher resistance rates, but more evidence to support this is necessary. The overall success rates of minocycline are in line with the existing literature. Similar success rates are reported in studies of VAP treated with combinations of β-lactams, or colistin or tigecycline, when the pathogen is susceptible to the administered regimen [53,54,55]. The mortality rate of the minocycline treated group was 20.9%. All deaths were associated with the primary infection and, therefore, they were considered as clinical failures of the minocycline-based regimen. Importantly, it is not reported by the authors whether these failures were attributable to the development of a minocycline resistance during the treatment or to the severity of the underlying infection. These data are highly important. There is a large debate on the mortality associated with *A. baumannii* infections; hence, the results of this review are again within the expected ranges [56,57]. Like the threat of plasmid mediated colistin resistance through the MCR-1 gene and the ongoing debate about increased mortality with tigecycline in critically ill ICU patients, the results of minocycline use reported here, are a welcome addition to our limited armamentarium and a possible trigger for further research [56,58,59,60,61,62,63].

The microbiological success rates in this study population were considerably lower than the relevant clinical rates. This may reflect the practical difficulties in pursuing follow-up samples in order to determine the bacterial eradication. Nevertheless, the role of *A. baumannii* as a colonizer cannot be overlooked. Many studies have demonstrated high prevalence rates in the colonization by resistant *A. baumannii* strains among immunocompromised patients with severe co-morbidities, like critically ill patients or residents of nursing homes [64,65,66,67]. These studies highlighted the epidemiologic profile of patients at risk of *A. baumannii* acquisition. Nevertheless, clinicians often face the dilemma of deciding when a bystander becomes a pathogen. Although in most cases the decision to treat is made upon individualized criteria, there is inadequate evidence to determine the transition of an isolate from a colonizer into a pathogen. The persistence of the pathogen in clinically non-significant counts has been reported in studies of VAP caused by *P. aeruginosa* and *A. baumannii*. This phenomenon may be attributed to the presence of biofilm-embedded bacterial populations, which are quite difficult to eradicate with currently achievable concentrations of antibiotics in deep lung tissues by the conventional intravenous route [68].

The growing issue of multidrug resistant *Acinetobacter* strains is firmly acknowledged, and the multifaceted consequences are now well-known to the medical community. This phenomenon is partially owed to the lack of large RCTs from the available literature. Minocycline in particular, although promising, has only been studied in observational studies. Therefore, the conduction of randomized trials, including minocycline in the evaluated regimens, along with other currently available agents, is imperative in order to create a solid base of evidence from which guidelines can be drawn. In an effort to address this difficult issue, a task force on the management of *A. baumannii* infections consisting of a multinational panel of experts, has recently issued a position paper on the management of *A. baumannii* infections in critically ill patients and particularly in the ICU setting [69]. Interestingly, monotherapy was recommended as a definite treatment of *A. baumannii* infections, irrespective of the source and type of the selected antibiotic, based on the lack of solid evidence supporting combination treatments as contributors to improved clinical outcomes [69]. According to the authors, “the combination of sulbactam or a polymyxin with a second agent (tigecycline, rifampicin, or fosfomycin) may be considered for clinical failures or for infections caused by an isolate with MIC in the upper limit of susceptibility” [69]. Minocycline is not recommended as a treatment option in this position paper [69].

Even with advanced antimicrobial regimens, MDR, XDR and PDR *A. baumannii* infections still carry high mortality rates and a considerable healthcare and economic burden. It seems that any treatment may be futile if the healthcare providers do not put efforts in impeding the spread of the *A. baumannii* strains. The more the clinicians administer extended-spectrum antibiotics to eliminate the increasingly resistant bacteria, the more “powerful” and resistant to available treatments those strains become. As currently there are no formulated, evidence-based guidelines that would direct not only the most active regimens, but also their doses and the duration of the treatment according to the site of the infection and the regional susceptibilities, the administered antibiotics may be suboptimal, thus inducing a vicious cycle of maintaining and enhancing the strains’ resistance. Consequently, instead of solely trying to treat them, the role of reducing the dissemination of these isolates within the healthcare environment should not be disregarded. To be pre-emptive is a rule of thumb in medicine, and in this case, the infection control measures are the way to achieve this. The European Society of Clinical Microbiology and Infectious Diseases (ESCMID) and the CDC have defined infection control measures for multidrug resistant bacteria, including MDR/XDR *Acinetobacter* [70,71]. These measures have shown very encouraging results in controlling and reducing the observed outbreaks in healthcare settings and, therefore, they should be established as common practice in nosocomial environments [72,73,74].

Multiple methodological challenges were faced in the present study and the results should be interpreted in the light of certain limitations. All included studies in this review were observational case series reporting minocycline use in small patient populations. This affects not only the statistical strength of the available data, but also the analysis and the interpretation of the results that were analyzed through an integrative manner that lacks the statistical strength of a meta-analysis. Unfortunately, there is a paucity of randomized controlled trials (RCTs) and the generalization of the results should therefore be done cautiously. Furthermore, the analyzed clinical studies demonstrate a notable diversity of selection criteria and patient characteristics. Unequivocally, this generates a sampling selection bias not only at the study level, but also at the level of cumulating evidence. Indeed, the retrospective researcher-directed recruitment of subjects on a minocycline-oriented therapy, without a comparator group in most cases, enhances the chance of a selection effect that may interfere with the observed clinical outcomes. Additionally, most studies performed in the USA reported the use of the intravenous (IV) formulation of minocycline (that was re-introduced there in 2009) [32]. However, the IV formulation is unavailable in many countries worldwide. Only recently, intravenous minocycline was introduced for licensing in the Latin America countries [75]. This may affect the applicability of the results in clinical practice, as there are no available studies comparing the oral versus the IV formulation in critically ill patients with gram-negative MDROs. Furthermore, the vast majority of the patients in this analysis received a minocycline combination rather than minocycline monotherapy. This raises the likelihood that the reported results and outcomes in the minocycline group may be influenced by the co-administered agent, thus resulting in confounding bias. Although the evidence is scarce for minocycline combinations, in clinical practice combination regimens are usually preferred over monotherapies in the treatment of resistant *A. baumannii* infections [50,76,77,78,79,80]. Another possible limitation of this review is the observed inconsistency between the definitions used for the isolates’ resistance among the selected trials. Indeed, only two studies (Goff et al. and Shi et al.) have defined the isolates in absolute concordance with the criteria set by established authorities dealing with antimicrobial resistance [30,34,38]. Even if not fully consistent with the synchronous terminology, these studies still fulfill the inclusion criteria as they involve strains that are at least MDR. All studies, except the one by Ning et al., evaluated strains that were either susceptible or with an intermediate susceptibility to minocycline [36]. However, only three included MIC data (Goff et al., Bishburg et al. and Pogue et al.) [32,34,37]. The susceptibility breakpoints were established using outdated methods that do not incorporate pharmacodynamics. There is growing evidence that broth microdilution (BMD) and VITEK 2 appear to be more reliable than an E-test for minocycline susceptibility testing in MDR *Acinetobacter spp.* [81].Variable susceptibility rates of MDR *Acinetobacter spp*., depending on the testing method, have been reported and MIC breakpoints may be lower than the ones currently used [82,83]. Furthermore, all included studies fail to report screening for the emergence of resistance to minocycline with repeat susceptibility testing. Interestingly, in the study by Chan et al.*,* an intermediate resistance was reported in 4 out of 6 patients receiving tigecycline [33]. Further efforts in examining the minocycline use should address the potential for the emergence of resistance or cross-resistance with other drugs of the same class. 

The mortality rate of MDR *A. baumannii* infections in this systematic review is considerably lower than previously reported data [14,15,16,17,18,19]. This bias is potentially introduced by the inclusion of less severe infections such as osteomyelitis and SSTIs. Additionally, the type of infection and the bioavailability may affect the activity of a static ribosomal targeting antibiotic like minocycline (BSI versus SSTI). Another major limitation was that **t**he time of the appropriate antimicrobial treatment initiation (including minocycline) was not addressed in the individual studies. Nevertheless, one could say that earlier antimicrobial therapy in some studies may have introduced a selection bias, thus favoring the use of minocycline. Finally, the included studies reported only scarce data on the safety profile of the drug.

Previously published reviews examining the role of minocycline in the treatment of resistant *A. baumannii* strains demonstrated similar results, under the constriction of the same limitations [7,76,84]. Nevertheless, the current systematic review includes more clinical studies and thus a larger patient population. Furthermore, it examines the role of both intravenous and oral formulations of minocycline in the treatment of resistant *A. baumannii* strains. Eligible studies were carefully examined in order to avoid overlapped populations. One study that has been included in previous reviews was therefore excluded from the analysis [34,85].

Taking into account its limitations, this review demonstrates evidence that minocycline may be an alternative therapy for nosocomial infections caused by resistant *A. baumannii* strains. Treatment options for MDR, XDR and PDR *A. baumannii* strains are considerably limited and inversely proportional to the resistance profile. Few antibiotics remain active and effective enough to endeavor a successful outcome in nosocomial infections caused by these strains. Among them, sulbactam, polymyxins, tigecycline and a combination of different classes of antibiotics are frequently used, with polymyxins being the current mainstay of treatment [24]. The medical community is in need of more therapeutic approaches, beyond colistin, as the exclusive administration and overuse of one antimicrobial class will inevitably limit the susceptible bacterial population, thus nullifying one more last-resort agent. Therefore, the evidence of using alternative therapeutic agents (alone or in combination) for *A. baumannii* infections like tigecycline, colistin, and sulbactam-based regimens should not be overlooked [86,87,88,89]. Minocycline is unique among the currently available therapeutic options against MDR *A. baumannii* in that it can be administered orally. This can be particularly important for clinically stable patients requiring a prolonged treatment.. Newer tetracycline derivatives may have an additional role in the future therapeutics of MDR *Acinetobacter* [90].

## 5. Conclusions

The prompt and optimal use of antibiotics is the solution of the perpetual puzzle of multi-resistant bacteria like *Acinetobacter*. However, what constitutes the optimal treatment still remains unresolved, while at the same time, the emergence of more resistant and virulent strains compels the need for an answer. Minocycline could constitute an alternative agent for the treatment of MDR *A. baumannii* infections. The therapeutic niche for minocycline could involve switching to oral therapy for clinically stable patients that require a prolonged treatment. However, the available clinical data that we reviewed, although promising, are still inconclusive. Research efforts with randomized studies, that utilize standard definitions for MDR/XDR/PDR organisms, employ uniform dosing regimens, evaluate the time of onset of an appropriate antimicrobial therapy and assess for the emergence of resistance by repeat susceptibility testing, should be made to define the role of currently available antibiotics, like minocycline, in the treatment of *A. baumannii* infections as novel agents are still under development or evaluation.

## Figures and Tables

**Table 1:** Definitions used in the systematic review.

Terms	Definitions
Clinical success- Clinical Cure	Elimination of the signs & symptoms related to the initial infection for which minocycline or non-minocycline based therapies are used.
Microbiological success- Microbiological Cure	Eradication of the causative organism from the site of infection.
Adverse Events	Onset of signs, symptoms or laboratory findings related to minocycline, its combinations or non-minocycline based regimens.
Hospital-Acquired Infection	The date of the site-specific infection occurs on or after the 3rd calendar day of admission *(the day of admission is calendar day 1)* [29].
Antimicrobial resistance classification	MDR: non-susceptible to ≥ 1 agent of ≥ 3 classes of antibiotics, XDR: non-susceptible to ≥ 1 agent in all but ≤2 categories, and PDR: non-susceptible to all classes or agents [30].
Site of infection	Site of isolation of the MDR, XDR or PDR *A. baumannii* (or *A. calcoaceticus-baumannii complex*) strain in the context of a new onset of relevant signs, symptoms and laboratory markers attributable to infection, based on the treating clinicians’ decision.
Surgical Site Infections	Infections that occur 30 days after surgery with no implant or within 1 year if an implant is placed and the infection appears to be related to surgery

MDR: multidrug resistant, XDR: extensively drug resistant, PDR: pandrug resistant.

**Table 2:** Included Clinical Studies.

Abbreviation ID of Study, Authors, Year, Reference	Type of Study/ Study Population (N)	Country, Region	Year of Study	Setting of Study	Purpose of Study
Study *A*, Bishburg E. et al., 2014 [32]	Retrospective Case Series (N = 21)	USA, Newark	11/2009–04/2012	Tertiary Care Hospital – Ward not specified.	To report the experience in using IV Minocycline for the treatment of MRSA and resistant gram-negative infections.
Study *B*, Chan J.D. et al., 2010 [33]	Retrospective Case Series (N = 55)	USA, Seattle	07/2004–12/2007	Trauma Centre – ICU.	To describe the clinical outcomes of case series of CR-AB VAP.
Study *C*, Goff D.A. et al., 2014 [34]	Retrospective Case Series (N = 55)	USA, Ohio	09/2010–03/2013	General Ward & ICU.	To describe an Antimicrobial Stewardship Program’s evaluation of Minocycline for the treatment of patients with MDR-AB infections.
Study *D*, Griffith M.E. et al.,2008 [35]	Retrospective Case Series (Retrospective Chart Review) (N = 8)	USA, Texas	2005–2006	Military Trauma Centre – Ward not specified.	To report the outcomes of patients received Minocycline as therapy for MDR-ABC traumatic wound infections.
Study *E*, Ning F. et al., 2014 [36]	Retrospective Case Series (N = 9)	CHINA Beijing	02/2011–03/2013	Department of Burn.	To report outcomes of treating extensive burns with PDR-AB infections with high doses of Meropenem, Cefoperazone –Sulbactam and Minocycline
Study *F*, Pogue J.M. et al., 2014 [37]	Retrospective Case Series (N = 9)	USA, Detroit	09/2011–n/a	Tertiary Care Hospital – Ward not specified.	To evaluate the use of IV minocycline for the treatment of CR-AB & *Enterobacteriaceae.*
Study *G*, Shi Y. et al., 2012 [38]	Prospective Single – Centre Trial (N = 77)	CHINA, Beijing	01/2009–12/2009	ICU	To explore the effects of cefoperazone/sulbactam plus minocycline on XDR-AB infections in critically ill patients.
Study *H*, Wang L.W. et al., 2014,[39]	Retrospective Case Series (N = 42)	CHINA, Beijing	04/2009–04/2010	Department of Respiratory Medicine/ICU.	To analyze the clinical features of PDR-AB and compare the efficacy of different antibiotic treatments in aged patients with PDR-AB VAP.
Study *I*, Wood G.C. et al., 2003 [40]	Retrospective Case Series (N = 7)	USA, Tennessee	01/1998–12/1998	Trauma Centre – ICU.	To report the use of tetracyclines for the treatment of MDR-AB VAP.
Study *J*, Vila A. et al., 2016 [41]	Retrospective Case Series (N = 3)	ARGENTINA, Mendoza	2010–2012	Tertiary Care Hospital – Ward not specified	To report 3 cases of MDR-AB prosthetic joint infections treated with debridement and tigecycline (2 patients received oral minocycline as maintenance treatment).

N refers to the total study population. AB: *A. baumannii*, ABC: *A. calcoaceticus-baumannii* complex, CR: Carbapenem resistant, ICU: Intensive care unit, IV: Intravenous, MDR: Multidrug resistant, MRSA: Methicillin–resistant *Staphylococcus aureus*, PDR: Pandrug resistant, USA: United States of America, VAP: Ventilator-associated pneumonia, XDR: Extensively drug resistant.

**Table 3:** Characteristics of included clinical studies.

Abbreviation ID of study, Authors, Year, Reference	Patient Demographics	*A. baumannii* Infections (*N =* Total No. of Patients)	No. of Patients on Minocycline (Monotherapy)	Organisms, Resistance Pattern Definition & Susceptibility Testing Method	Minocycline Route of Administration & Doses	Minocycline Combinations	Other Antimicrobial Agents & Doses	Clinical Success Definition	Microbiological Success Definition
Study *A*, Bishburg E. et al., 2014 [32]	Male: 10, Female: 11 Age Range: 25–83 y.o. (*n/a separate data for patients with AB infections*)	BSI (*n* = 1), SSTI (*n* = 2), LRTI (*n* = 2) (***N*** = **5**)	5 (n/a)	MDR-AB Not defined E-test	100 mg I.V. B.I.D., converted to P.O. per physician decision	*Not applicable*	*Not applicable*	Not defined.	Not defined.
Study *B*, Chan J.D.et al., 2010 [33]	Male: 40, Female: 15 Age Range: 18–87 y.o.	VAP (***N*** = **55**)	36 (11)	CR-AB Not defined. Not reported.	200 mg LD followed by 100 mg I.V. B.I.D. *OR* 200 mg P.O. B.I.D.	AMG(*n* = 20), AMG + TGC (*n* = 3), AMG + PLM (*n* = 2)	TGC: 100 mg LD – then 50 mg I.V. B.I.D., TOB: 7 mg/kg I.V. OD. *OR* 300 mg INH B.I.D., GEN: 7 mg/kg I.V. OD, AMK: 15 mg/kg I.V. OD, PLM-B: 2.5–5 mg/kg per day B.I.D. or Q.I.D., COL: 2.5–5 mg/kg per day B.I.D. or Q.I.D. or 150 mg INH B.I.D., SAM: 2+1 gr I.V. Q.I.D.	Improvement and resolution of signs and symptoms of VAP.	Eradication of CR-AB from subsequent BAL or sputum culture at the completion of therapy.
Study *C*, Goff D.A. et al., 2014 [34]	Male: 36, Female: 19 Age Range: 23–85 y.o.	LRTI (*n* = 32), BSI (*n* = 10), IAI (*n* = 3), LRTI+BSI (*n* = 4), SSTI (*n* = 2),Osteomyelitis (*n* = 2), UTI (*n* = 2) (***N*** = **55**)	55 (3)	MDR-AB Non-susceptible to ≥1 antibiotic agent in ≥3 categories. E-test	100 mg I.V. B.I.D. (*n* = 42 received 200mg I.V. LD)	COL I.V. (*n* = 19) DOR + COL I.V. (*n* = 9), SAM+COL I.V. (n = 7), DOR+COL INH (*n* = 4), SAM (*n* = 3) SAM + DOR + COL I.V. (*n* = 3) DOR (*n* = 2) SAM + DOR (*n* = 2) SAM + COL INH (*n* = 1), COL INH (*n* = 1) SAM + COL I.V.+ COL INH (*n* = 1)	*Not applicable*	Complete or partial resolution of the signs & symptoms attributable to the MDR-AB infection without a need for the escalation of antimicrobials.	Eradication of MDR-AB in follow-up cultures from the primary source of infection during treatment *or* presumed when patient clinically improved & follow-up cultures were not performed.
Study *D*, Griffith M.E. et al., 2008 [35]	Male: 8 Age Range: 19–35 y.o.	Osteomyelitis & SSTI (***N*** = **8**)	8 (1)	MDR-ACB Resistance to all commonly tested Cephalosporins, β-Lactam /β-Lactamase Inhibitor & Fluoroquinolones. Disk Diffusion/BMD	100 mg P.O. B.I.D.	VAN (*n* = 2), AMK (*n* = 1) IPM (*n* = 4)	*Not applicable*	No further clinical evidence of infection as determined by symptoms, physical examination, laboratory evaluation.	Not defined
Study *E*, Ning F.et al., 2014 [36]	Male: 6, Female: 3 Age Range: 23–57 y.o.	LRTI (*n* = 3), LRTI + SSTI (*n* = 2), LRTI + SSTI + BSI (*n* = 4) (***N*** = **9**)	9 (0)	PDR-AB Resistant to all antibiotics except PLM B. Not reported	200 mg P.O. Q.I.D.	MEM I.V. Q.I.D. (6gr/day total).+CFP/S 6 gr I.V B.I.D.	*Not applicable*	Drugs were withdrawn after symptoms’ improvement combined with negative blood & sputum cultures.	Negative blood and sputum cultures.
Study*F*, Pogue J.M. et al., 2014 [37]	Male: 5, Female: 2 Age Range: 35–74 y.o.	BSI (*n* = 3), LRTI (*n* = 3), LRTI + SSTI (*n* = 1) (***N*** = **7**)	7 (1)	CR-AB Resistant to all CRB. E-test	100 mg I.V. B.I.D. (*n* = 2) 200 mg I.V. B.I.D. (*n* = 5)	MEM + COL (*n* = 1), COL (*n* = 4), SAM (*n* = 1)	*Not applicable*	Resolution of signs & symptoms of the infection requiring MIN.	Clearance of the organism of interest from repeated cultures.
Study *G*, Shi Y. et al., 2012 [38]	Male: 49, Female: 28 Age Range: 49–89 y.o.	LRTI (*n* = 61), BSI (*n* = 5), Intra-abdominal (*n* = 3), SSTI (*n* = 2), Multiple sites (*n* = 6) (***N*** = **77**)	77 (0)	XDR-AB Sensitive only to 1 or 2 classes of antibiotics. n/a	100 mg I.V. or P.O. B.I.D.	CFP/S (*n* = 70) CFP/S + IPM/CIL (*n* = 7)	*Not applicable*	Clinical effect divided in: cure, marked improvement, improvement & ineffectiveness. Effective group defined as cure+marked improvement.	Cleared cultures.
Study *H*, Wang L.W. et al., 2014 [39]	Mean age: 89.1+/−3.2	VAP (***N*** = **42**)	20 (0)	PDR-AB Not defined. n/a	n/a	CFP/S	n/a	Not defined	Bacterial eradication of PDR-AB from cultures.
Study *I*, Wood G.C. et al., 2003 [40]	Male: 7 (age n/a)	VAP (*n* = 5), VAP + BSI (*n* = 1), VAP + SSTI (*n* = 1) (***N*** = **7**)	4 (2)	MDR-AB Resistance to all antibiotics including IPM-CIL and SAM. VITEK	100 mg I.V. B.I.D.	IPM-CIL (*n* = 1) TVA + TMP/SMX (*n* = 1).	DOX 100 mg I.V. B.I.D.	AB was absent from the follow-up BAL cultures and the patient improved clinically ***OR*** The patient improved clinically& survived until discharge.	AB was absent from the follow-up BAL cultures.
Study *J*, Vila A. et al., 2016 [41]	Male: 1, Female: 2 Age Range: 45–75 y.o.	PJI (*n* = 3) (***N*** = 3)	2 (0)	MDR-AB Non- susceptible to ≥3 categories. Disk diffusion	200 mg P.O/day	TGC + COL (prior to oral minocycline) (*n* = 2)	TGC: 100 mg LD – then 100 mg I.V. B.I.D, COL	No signs and symptoms of infection, CRP < 10 mg/L, normal ESR, absence of radiological signs of loosening at the end of treatment, without further recurrences.	Negative tissue cultures from subsequent debridements.

AB: *Acinetobacter baumannii*, ACB: *Acinetobacter calcoaceticus-baumannii complex*, AMG: Aminoglycoside, AMK: Amikacin, BAL: Bronchoalveolar lavage, B.I.D: twice a day, BMD: Broth microdilution, BSI: Bloodstream Infection, CFP/S: Cefoperazone/Sulbactam, CIL: Cilastatin, COL: Colistin, CR: Carbapenem resistant, CRB: Carbapenem, CRP: C – reactive protein, DOR: Doripenem, DOX: Doxycycline, ESR: Erythrocyte sedimentation rate, gr: grams, GEN: Gentamycin, IAI: Intra-abdominal infection, INH: Inhaled, IPM: Imipenem, I.V.: Intravenous, kg: Kilograms, LD: Loading dose, LRTI: Lower respiratory tract infection, MDR: Multidrug resistant, MEM: Meropenem, mg: milligrams, MIN: Minocycline, n/a: not available data, OD: once daily, PDR: Pandrug resistant, PLM: Polymyxin, PLMB: Polymyxin B, P.O.: orally, PJI: Prosthetic joint infection, Q.I.D: four times a day, SAM: Ampicillin/Sulbactam, SSTI: Skin and soft tissue infection, TGC: Tigecycline, TOB: Tobramycin, TMP/SMX: Trimethoprim/Sulfamethoxazole, TVA: Trovafloxacin, UTI: Urinary tract infection, VAN: Vancomycin, VAP: Ventilator-associated pneumonia, XDR: Extensively drug resistant, y.o.: years old.

**Table 4:** Outcomes of included clinical studies.

Abbreviation ID of Study, Authors, Year, Reference	Clinical Success		Microbiological Success		Mortality		Adverse Events	
	Minocycline (%)	Other Antibiotics (%)	Minocycline (%)	Other Antibiotics (%)	Minocycline (%)	Other Antibiotics (%)	Minocycline (%)	Other Antibiotics (%)
Study *A*, Bishburg E. et al., 2014 [32]	5 (100) *BSI: 1 (100)LRTI: 2 (100)*	n/a	*Not available*	n/a	0	n/a	*Not available*	n/a
Study *B*, Chan J.D. et al., 2010[33]	29 (80.5) *VAP: 29 (80.5)*	13 (68.4)	Not separately specified *	Not separately specified *	Not separately specified *	Not separately specified *	0	Nephrotoxicity due to aminoglycosides or polymyxins: 10 (18.2)
Study *C*, Goff D.A. et al., 2014 [34]	40 (72.7) *LRTI: 20 (62.5)* ^§^ *BSI: 9 (90)* ^§^ *LRTI+BSI: 3 (75)* ^§^	n/a	43 (78) ^±^	n/a	14 (25.5)	n/a	0	n/a
Study *D*, Griffith M.E. et al., 2008 [35]	7 (87.5)	n/a	*Not available*	n/a	0	n/a	Eosinophilia & neutropenia: 1 (12.5)	n/a
Study *E*, Ning F. et al., 2014 [36]	9 (100) *LRTI: 3 (100) LRTI + SSTI: 2 (100) LRTI + SSTI + BSI: 4 (100)*	n/a	8 (88.9) ^±^	n/a	0	n/a	0	n/a
Study *F*, Pogue J.M. et al., 2014 [37]	5 (71.4) *LRTI: 1 (50)* *BSI: 2 (66.6)* *LRTI + SSTI: 1 (100)* *LRTI + BSI: 1 (100)*	n/a	3 (60) ** LRTI + BSI: 1 (100) *BSI: 2 (66.6)*	n/a	2 (28.6)	n/a	Nephrotoxicity due to colistin combination: 2 (28.6)	n/a
Study *G*, Shi Y. et al., 2012 [38]	48 (62.3) ^±^	n/a	36 (46.8) ^±^	n/a	19 (24.7)	n/a	Elevation of LFTs: 6 (7.8)	n/a
Study *H*, Wang L.W. et al., 2014 [39]	13 (65) *VAP: 13 (65)*	5 (22.7) *VAP: 5 (22.7)*	8 (40) *VAP: 8 (40)*	3 (13.6) *VAP: 3 (13.6)*	*Not available*	*Not available*	*Not available*	*Not available*
Study *I*, Wood G.C. et al., 2003 [40]	4 (100) *VAP: 4 (100)* ^±^	2 (66.7) *VAP:* 2 (66.7) ^±^	3 (100) ** VAP: 3 (100) ^±^	1 (50) ** VAP: 1 (50) ^±^	0	1 (33.3)	0	0
Study *J*, Vila A. et al., 2016 [41]	2 (100)	1 (100)	2 (100)	1 (100)	0	0	0	0

* Data were not available for “Minocycline group” and “Other Antibiotics” group separately. ** Percentages were calculated for patients with available data. ^§^ Data were not available for 1 patient with clinical failure. ^±^ Data were not available for all types of infections separately. BSI: Bloodstream Infection, LFTs: Liver function tests, LRTI: Lower respiratory tract infection, n/a: not applicable data, SSTI: Skin and soft tissue infection, VAP: Ventilator-associated pneumonia.

## Supplementary Materials

The following is available online at https://www.mdpi.com/2076-2607/7/6/159/s1: Table S1: The PRISMA-P2015 Checklist.

## Appendix A

**Table A1:** The STROBE statement checklist scores for included studies.

Abbreviation ID of study, Authors, Year, Reference	Satisfied Criteria of the STROBE Checklist	Score
Study *A*, Bishburg E. et al., 2014 [32]	1(a,b),2,3,4,5,6(a),7,8,10,11,13(a),14(a),15,18,19,20,21,22	18/22
Study *B*, Chan J.D. et al., 2010 [33]	1(a,b),2,3,4,5,6(a),7,8,10,11,13(a),14(a),15,17,18,19,20,21,22	19/22
Study *C*, Goff D.A. et al.*,* 2014 [34]	1(a,b),2,3,4,5,6(a),7,8,10,11,13(a),14(a),15,17,18,19,20,21,22	19/22
Study *D*, Griffith M.E. et al., 2008 [35]	1(a,b),2,3,4,5,6a,7,8,10,11,13a,14(a,b),15,17,18,19,20,21,22	19/22
Study *E*, Ning F.et al., 2014 [36]	1(a,b),2,3,4,5,6(a),7,8,10,11,12(a,b),13(a),14(a,b),15,17,18,19,20,21,22	20/22
Study *F*, Pogue J.M. et al., 2014 [37]	1(a,b),2,3,4,5,6(a),7,8,10,11,13(a,b),14(a,b),15,16(a),17,18,19,20,21,22	20/22
Study *G*, Shi Y. et al., 2012 [38]	1(a,b),2,3,4,5,6,7,8,9,10,11,12(a,b),13(a),14(a,b),15,16(a),17,18,19,20,21,22	22/22
Study *H*, Wang L.W. et al., 2014 [39]	1(a,b),2,3,4,5,6(a),7,8,10,11,13(a),14(a),15,18,19,20,21,22	18/22
Study *I,* Wood G.C. et al., 2003 [40]	1(a,b),2,3,4,5,6,7,8,10,13(a,b),14(a,b),15,17,18,19.20,21,22	18/22
Study*J*, Vila A. et al., 2016 [41]	1(a,b),2,3,4,5,6a,7,8,10,11,13a,14(a,b),15,17,18,19,20,21,22	19/22
